# Long-term outcomes and prognostic factors of hybrid aortic arch repair: a 15-year cohort study

**DOI:** 10.3389/fcvm.2026.1933618

**Published:** 2026-09-04

**Authors:** Guanqing Li, Aijun Chen, Ting Yan, Haisheng Chen, Bin Li, Dongting Ye, Daliang Zhu, Xiaoran Guan, Jianrong Zhou, Xiong Zhang

**Affiliations:** 1 Department of Cardiovascular Surgery, Guangzhou First People’s Hospital, School of Medicine, South China University of Technology, Guangzhou, China; 2 Paediatric Cardiac Surgery Centre, National Clinical Research Centre for Cardiovascular Diseases, State Key Laboratory of Cardiovascular Disease, Fuwai Hospital, Chinese Academy of Medical Sciences & Peking Union Medical College/National Centre for Cardiovascular Diseases, Beijing, China

**Keywords:** aortic arch lesions, Cox regression, hybrid aortic arch repair, long-term outcomes, survival analysis, thoracic endovascular aortic repair

## Abstract

**Objective:**

To evaluate long-term outcomes of hybrid aortic arch repair for aortic arch lesions and identify factors associated with late mortality.

**Methods:**

We retrospectively enrolled 103 patients with aortic arch lesions who received hybrid aortic arch repair at our center between January 2010 and September 2025. General clinical data, lesion types, surgical methods, perioperative outcomes, and long-term follow-up data were collected. Based on the bypass strategy, patients were divided into an ascending aorta (AA) inflow group (*n* = 48) and a cervical debranching group (*n* = 55). Kaplan–Meier analysis and Cox proportional hazards regression were used to evaluate long-term outcomes and identify factors associated with late mortality.

**Results:**

The mean age was 72.0 ± 10.2 years, and 83.5% were men. Diagnoses included intramural hematoma (40.8%), type B dissection (17.5%), type A dissection (14.6%), arch aneurysm (14.6%), and penetrating ulcer (12.6%). All patients successfully completed the procedure, with no intraoperative or 30-day deaths. During a median follow-up of 75.8 months (IQR, 29–128), procedure-related stroke occurred in 8 patients (7.8%), renal dysfunction in 18 (17.5%), and spinal cord ischemia in none. Eleven patients (10.7%) died, and 8 (7.8%) underwent reintervention, mainly for retrograde type A dissection, endoleak, or new-entry dilation. Survival at 1, 3, 5, and 10 years was 98.0%, 94.6%, 89.0%, and 85.8%; freedom from reintervention was 97.9%, 94.2%, 91.5%, and 89.9%. Cervical debranching was associated with better survival than ascending aortic inflow (log-rank *P* = 0.024), with similar freedom from reintervention (log-rank *P* = 0.88). In an exploratory multivariable Cox model, older age, ascending aorta inflow, and preoperative renal dysfunction were associated with late mortality.

**Conclusions:**

Hybrid aortic arch repair was associated with favorable long-term survival, durable freedom from reintervention, and acceptable neurological outcomes in this single-center cohort. The observed survival advantage in the cervical debranching group should be interpreted cautiously, as ascending aorta inflow was preferentially used in patients with more complex proximal aortic disease. These findings should be considered hypothesis-generating and require validation in larger multicenter studies.

## 1 Introduction

Aortic arch lesions, including aortic dissection, aneurysm, intramural hematoma, and penetrating aortic ulcer, are potentially life-threatening cardiovascular conditions associated with substantial morbidity and mortality (1). Because of the complex anatomy of the aortic arch and its proximity to critical branch vessels, including the innominate artery, common carotid artery, and subclavian artery, conventional open total arch replacement frequently requires cardiopulmonary bypass, deep hypothermic circulatory arrest, and complex cerebral protection strategies. These factors increase perioperative risk and may limit the applicability of open repair in elderly patients and those with multiple comorbidities (2–5).

With the continuous evolution of thoracic endovascular aortic repair (TEVAR) and perioperative management, total endovascular approaches using branched, fenestrated, or physician-modified devices have emerged as alternatives for selected patients with aortic arch disease. These techniques may further reduce surgical trauma and avoid cervical or sternotomy-based debranching. However, their use remains limited by anatomical suitability, device availability, regulatory approval, technical complexity, and uncertain long-term durability. Therefore, hybrid aortic arch repair (HAR) continues to play an important role, particularly in centers where branched arch devices are not widely available or in patients requiring individualized landing-zone creation. HAR achieves supra-aortic branch debranching or bypass reconstruction through an open approach, followed by TEVAR to establish an effective proximal landing zone and seal the lesion. To a certain extent, this balances the reliability of open reconstruction with the minimally invasive advantages of endovascular treatment (6–10). Current literature indicates that HAR yields favorable early and mid-term outcomes in high-risk patients. However, significant heterogeneity remains regarding long-term results, reintervention risks, and the impact of different surgical strategies on late prognosis (11–13).

Recently, studies focusing on the long-term outcomes of HAR have increased. The 5-year and 10-year survival rates reported by different centers vary considerably, and the identified factors affecting long-term prognosis remain inconsistent. Previous reports suggest that age, lesion type, landing zone, history of reoperation, and surgical approach may all influence the late outcomes of HAR (7, 12, 14–18). Nevertheless, due to variations in patient demographics, disease complexity, procedural stratification, and institutional experience, these conclusions still require further supplementation and validation through long-term follow-up data from single centers.

Therefore, this study retrospectively analyzed the clinical data and long-term follow-up results of patients treated with HAR for aortic arch lesions at our center. By evaluating their overall survival, freedom from reintervention, and major complications, and by further analyzing the factors influencing the risk of long-term mortality, we aim to provide a clinical reference for surgical selection and postoperative management.

## 2 Methods

### 2.1 Study population

We retrospectively reviewed patients who underwent HAR for aortic arch lesions at our center between January 2010 and September 2025. The inclusion criteria were as follows: (1) a definitive diagnosis of an aortic arch lesion confirmed by computed tomography angiography; (2) treatment with HAR; and (3) availability of complete perioperative and long-term follow-up data. The exclusion criteria were: (1) emergent HAR for ruptured aortic arch lesions; and (2) insufficient follow-up data. The study protocol was approved by the Ethics Committee of Guangzhou First People's Hospital (No. K-2026-174-01). Given the retrospective nature of the study, the requirement for informed consent was waived.

### 2.2 Data collection

Patient information was extracted via the electronic medical record system, surgical notes, imaging archives, and follow-up data. The collected study variables included: general clinical characteristics (such as age, gender, comorbidities, history of median sternotomy), primary pathological diagnosis, surgical techniques, perioperative outcomes, and long-term endpoints (death, reintervention, and related complications).

### 2.3 Surgical strategies

The debranching configuration was selected according to the intended proximal landing zone, the supra-aortic branches requiring coverage, donor and recipient vessel quality and diameter, vertebral artery dominance, and involvement of the left common carotid artery. For Zone 2 TEVAR with isolated left subclavian artery coverage, left common carotid artery–left subclavian artery (LCCA-LSA) bypass was generally preferred. If the left common carotid artery was unsuitable as a donor vessel because of severe calcification, inadequate diameter, or unfavorable left cervical anatomy, right subclavian artery–left subclavian artery (RSA-LSA) bypass was considered. For Zone 1 TEVAR requiring coverage of both the left common carotid artery and left subclavian artery, right subclavian artery–left common carotid artery–left subclavian artery (RSA-LCCA-LSA) bypass or right common carotid artery–left common carotid artery–left subclavian artery (RCCA-LCCA-LSA) bypass was selected according to cervical vascular anatomy. For Zone 0 TEVAR or lesions involving the ascending aorta (AA) or proximal arch, AA inflow with multi-branch supra-aortic reconstruction was performed. When significant ascending aortic disease was present, concomitant ascending aortic replacement or Bentall procedure was performed as clinically indicated. All procedures were performed in a hybrid operating room equipped with fixed angiography systems.

Based on the structural differences in supra-aortic debranching, patients were stratified into two groups: the ascending aortic inflow group and the cervical debranching group.

Ascending aortic inflow group: For patients requiring Zone 0 anchoring or those with concurrent ascending aortic pathologies (such as type A aortic dissection), a median sternotomy was performed. The bypass graft was anastomosed directly to the ascending aorta to obtain inflow, such as ascending aorta-innominate artery-left common carotid artery-left subclavian artery bypass (AA-IA-LCCA-LSA). In patients with extensive ascending aortic involvement, an ascending aortic replacement or Bentall procedure was performed concurrently with the debranching, followed by antegrade or retrograde implantation of the TEVAR stent-graft.

Cervical Debranching Group: For patients with a suitable proximal ascending aorta (typically Zone 1 or Zone 2 anchoring for TEVAR), extra-anatomic bypass grafts (e.g., RSA-LSA, LCCA-LSA, or RSA-LCCA-LSA bypass) were performed exclusively via cervical incisions, avoiding a median sternotomy. Following debranching, a TEVAR stent-graft was deployed retrogradely via the femoral artery to isolate the arch lesion.

In this study, either 8-mm ringed polytetrafluoroethylene (PTFE) or woven Dacron grafts were used. Meticulous care was taken to ensure tension-free and kink-free configurations during anastomosis.

### 2.4 Follow-up and endpoint definitions

Postoperative surveillance included clinical evaluations and computed tomography angiography scans, conducted prior to discharge, at 3, 6, and 12 months postoperatively, and annually thereafter. The primary endpoint was all-cause mortality. Secondary endpoints encompassed reintervention, graft stenosis or occlusion, endoleak, stent migration or structural failure, stroke, spinal cord ischemia, and renal dysfunction. Preoperative renal dysfunction was defined as an estimated glomerular filtration rate <60 mL/min/1.73 m^2^ or a documented history of chronic kidney disease. Postoperative renal dysfunction was defined according to KDIGO criteria (19) as an increase in serum creatinine by ≥0.3 mg/dL within 48 h or ≥1.5 times baseline within 7 days after the procedure. Overall survival was calculated from the date of discharge to the date of death or last follow-up. Time to reintervention was defined from discharge to the date of the re-do procedure, with patients free from reintervention censored at their last follow-up. Procedure-related stroke was defined as any new focal neurological deficit confirmed by cranial computed tomography or magnetic resonance imaging occurring within 30 days after HAR or before discharge, and judged by neurologists to be ischemic or embolic in origin.

### 2.5 Statistical analysis

Continuous variables were summarized as means ± standard deviations or medians with interquartile ranges (IQR), as appropriate, and compared using independent-sample *t*-tests or Mann–Whitney *U* tests, as appropriate. Categorical variables were described by frequencies and percentages, and analyzed using either the Pearson chi-square or Fisher's exact test. For survival outcomes, we estimated overall survival and freedom from reintervention using the Kaplan–Meier method and compared group differences with the log-rank test. To identify predictors of long-term mortality, we first performed a univariate Cox proportional hazards analysis. Any variable showing statistical significance was subsequently included in a multivariate Cox regression model. The resulting hazard ratios (HR) and their 95% confidence intervals (CI) were documented. The proportional hazards assumption was successfully verified via Schoenfeld residuals. Data analyses were conducted using SPSS 28.0 and R, considering a two-sided *P*-value of less than 0.05 as statistically significant.

## 3 Results

### 3.1 Baseline clinical characteristics

The baseline characteristics of the 103 patients are summarized in Table 1. The mean age was 72.0 ± 10.2 years, and most patients were men (83.5%). The most common primary diagnosis was intramural hematoma (40.8%), followed by type B aortic dissection (17.5%) and type A aortic dissection (14.6%). Patients in the ascending aortic inflow group were significantly younger than those in the cervical debranching group (69.3 ± 8.4 vs. 74.5 ± 10.6 years, *P* = 0.006). Notably, all patients with type A aortic dissection were in the ascending aortic inflow group, suggesting substantial differences in baseline disease complexity between the two groups (*P* < 0.001). The distributions of comorbidities were not significantly different between groups.

**Table 1:** Baseline clinical characteristics of the patients.

Variables	Total (*N* = 103)	Cervical debranching group (*n* = 55)	Ascending aorta inflow group (*n* = 48)	*P-*value
Age (years)	72.0 ± 10.2	74.5 ± 10.6	69.3 ± 8.4	0.006*
Male, *n* (%)	86 (83.5)	45 (81.8)	41 (85.4)	0.624
Comorbidities, *n* (%)				
Hypertension	92 (89.3)	47 (85.5)	45 (93.8)	0.174
Diabetes mellitus	56 (54.3)	30 (54.5)	26 (54.2)	0.881
Hyperlipidemia	72 (69.9)	38 (69.1)	34 (70.8)	0.852
Coronary heart disease	34 (33.0)	16 (29.1)	18 (37.5)	0.363
Hepatic dysfunction	2 (1.9)	1 (1.8)	1 (2.1)	1.000
Preoperative renal dysfunction	7 (6.8)	3 (5.5)	4 (8.3)	0.460
Previous sternotomy	8 (7.8)	3 (5.5)	5 (10.4)	0.293
Primary diagnosis, *n* (%)				<0.001
Type A aortic dissection	15 (14.6)	0 (0.0)	15 (31.3)	
Type B aortic dissection	18 (17.4)	13 (23.6)	5 (10.4)	
Aortic arch aneurysm	15 (14.6)	9 (16.4)	6 (12.5)	
Intramural hematoma	42 (40.8)	25 (45.5)	17 (35.4)	
Penetrating aortic ulcer	13 (12.6)	8 (14.5)	5 (10.4)	

Continuous variables are expressed as mean ± standard deviation (SD), and categorical variables are expressed as frequency (percentage). For continuous variables with normal distribution, an independent samples *t*-test was used. For categorical variables, the Pearson Chi-square test was used; Fisher's exact test was applied when the expected frequency in any cell was <5. A two-sided *P* < 0.05 was considered statistically significant.

The symbol (*) indicates statistically significant results (*P* < 0.05).

### 3.2 TEVAR devices used during the study period

The thoracic endografts used during the 15-year study period varied over time. In the early phase of the cohort, several commercially available devices were used, including Medtronic (e.g., Valiant™), Gore (e.g., GORE® TAG®/Conformable TAG®), JOTEC (e.g., E-vita Thoracic), Cook (e.g., Zenith® Alpha™/TX2®), Lifetech Scientific (e.g., Ankura™), and MicroPort (e.g., Hercules™/Castor™) stent-grafts. With increasing institutional experience and changes in device availability, later practice gradually shifted toward a more limited range of devices, mainly Gore, MicroPort, and Lifetech Scientific endografts. Device selection was based on aortic diameter, arch angulation, landing-zone length, access vessel anatomy, delivery system characteristics, availability, and operator preference. JOTEC and Cook devices were used relatively infrequently in our center, partly because of limited domestic use and device-specific anatomical applicability. Medtronic devices were used in selected cases, although their use was limited by the available device options during the study period. Details of the TEVAR devices used are provided in Supplementary Table 1.

### 3.3 Surgical details and perioperative outcomes

A variety of supra-aortic debranching configurations were employed in this cohort (Table 2). The most prevalent procedures were RSA-LSA bypass (21.4%). Twelve patients (11.6%) underwent concurrent ascending aortic replacement, and 3 (2.9%) underwent a Bentall procedure. There were no intraoperative or 30-day postoperative deaths.

**Table 2:** Surgical procedures of prosthetic bypass.

Surgical procedures	*n*	%
Cervical debranching group	55	53.4
RSA-LSA	22	21.4
LCCA-LSA	17	16.5
RSA-LCCA-LSA	10	9.7
RCCA-LCCA-LSA	6	5.8
Ascending aorta inflow group	48	46.6
AA-IA-LCCA	18	17.5
AA-IA-LCCA-LSA	15	14.6
Ascending aortic replacement+AA-IA-LCCA-LSA	12	11.6
Bentall procedure+AA-IA, AA-LCCA, AA-LSA	3	2.9
Total	103	100.0

AA, ascending aorta; IA, innominate artery; LCCA, left common carotid artery; LSA, left subclavian artery; RCCA, right common carotid artery; RSA, right subclavian artery.

As shown in Table 3, the mean postoperative systolic blood pressure difference between arms was 17.3 ± 4.1 mmHg, and more than 90% of patients had an inter-arm pressure gradient of <20 mmHg. Procedure-related stroke occurred in 8 patients (7.8%). All events developed during the index hospitalization, with a median onset of 10 days after surgery. Among these patients, 7 achieved partial neurological recovery, whereas 1 had persistent neurological deficit. No deaths were attributed to stroke-related complications. Stroke occurring beyond 30 days after surgery were not considered procedure-related stroke. No patient developed spinal cord ischemia. Postoperative renal dysfunction occurred in 18 patients (17.5%). Of these, 11 experienced transient acute kidney injury and recovered before discharge, 4 had persistent renal impairment at discharge, and 3 required temporary dialysis for a median duration of 5 days. No patient required permanent dialysis.

**Table 3:** Perioperative outcomes and outcomes during follow-up after hybrid procedures.

Variables	Total (*N* = 103)	Cervical debranching group (*n* = 55)	Ascending aorta inflow group (*n* = 48)	*P*-value
Perioperative outcomes				
30-day mortality, *n* (%)	0 (0.0)	0 (0.0)	0 (0.0)	-
Procedure-related stroke, *n* (%)	8 (7.8)	5 (9.1)	3 (6.3)	0.718
Procedure-related spinal cord ischemia, *n* (%)	0 (0.0)	0 (0.0)	0 (0.0)	-
Procedure-related renal dysfunction, *n* (%)	18 (17.5)	10 (18.2)	8 (16.7)	0.840
Outcomes during follow-up				
Follow-up duration (months)	75.8 (29.0–128.0)	78.0 (28.1–134.0)	75.1 (30.5–125.0)	0.890
Mortality during follow-up, *n* (%)	11 (10.7)	2 (3.6)	9 (18.8)	0.021*
Reoperation, *n* (%)	8 (7.8)	4 (7.3)	4 (8.3)	1.000
Prosthetic graft stenosis or occlusion, *n* (%)	2 (1.9)	1 (1.8)	1 (2.1)	1.000
Endoleak, *n* (%)	1 (1.0)	1 (1.8)	0 (0.0)	1.000
New or dilated entry tear, *n* (%)	3 (2.9)	2 (3.6)	1 (2.1)	1.000
Retrograde aortic dissection, *n* (%)	2 (1.9)	1 (1.8)	1 (2.1)	1.000
Psychiatric disorders (e.g., depression), *n* (%)	2 (1.9)	0 (0.0)	2 (4.2)	0.215
Postoperative systolic blood pressure difference between arms (mmHg)	17.3 ± 4.1	16.8 ± 4.9	17.9 ± 3.7	0.492

Data are presented as *n* (%), mean ± standard deviation, or median (interquartile range, IQR). *P*-values were calculated using Student's *t*-test for normally distributed continuous variables, the Mann–Whitney *U* test for non-normally distributed continuous variables (e.g., follow-up duration), and the Chi-square or Fisher's exact test for categorical variables, as appropriate. A two-sided *P* < 0.05 was considered statistically significant. (-) indicates that the *P*-value was not calculated because no events occurred in either group.

The symbol (*) indicates statistically significant results (*P* < 0.05).

### 3.4 Long-term follow-up and clinical outcomes

**Figure 1:**
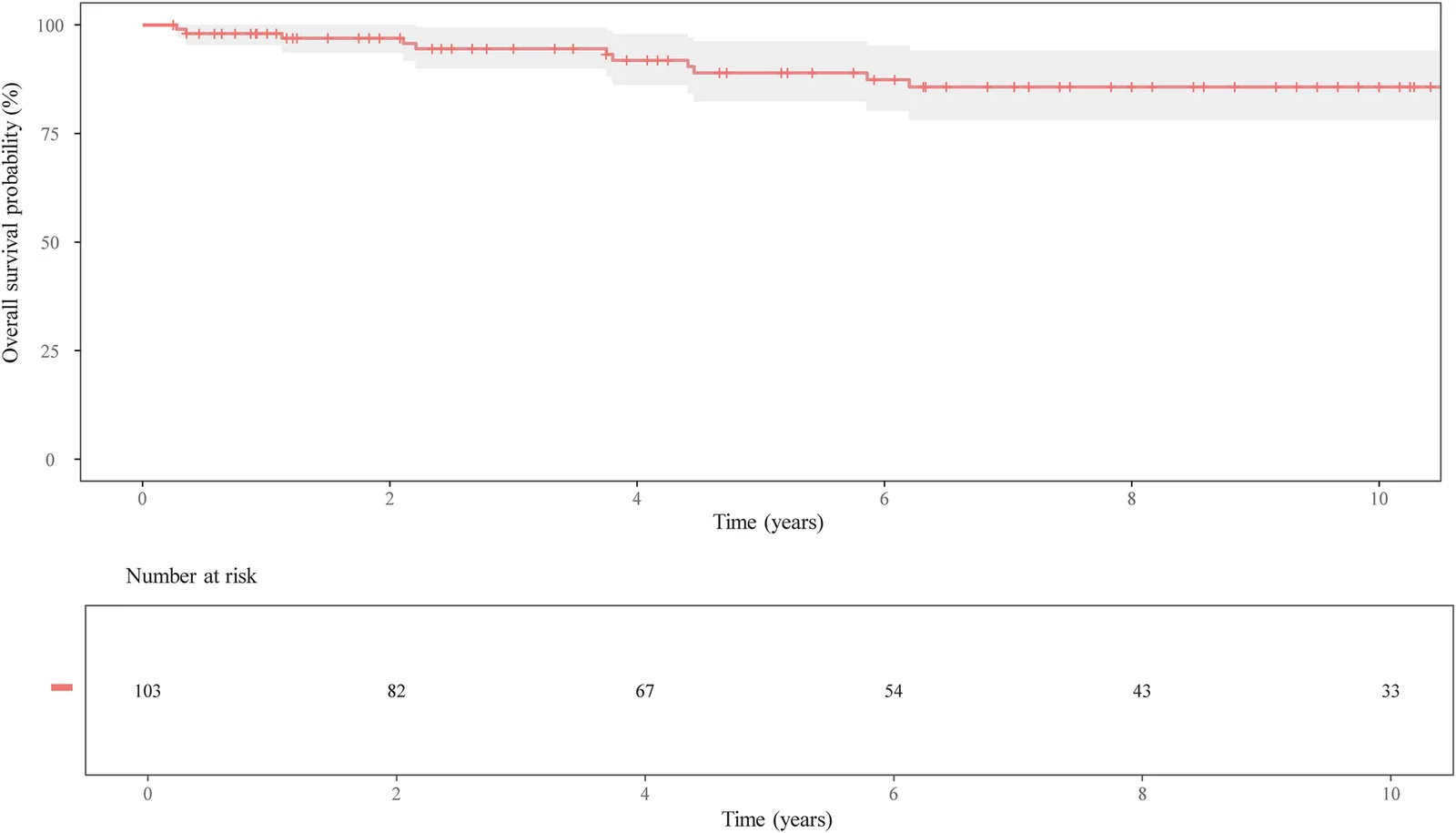
Kaplan–Meier overall survival curve for the entire cohort after hybrid aortic arch repair. Kaplan–Meier analysis showing overall survival in the entire cohort of 103 patients who underwent hybrid aortic arch repair for aortic arch pathologies. The shaded area represents the 95% confidence interval. Numbers at risk are shown below the *x*-axis.

**Figure 2:**
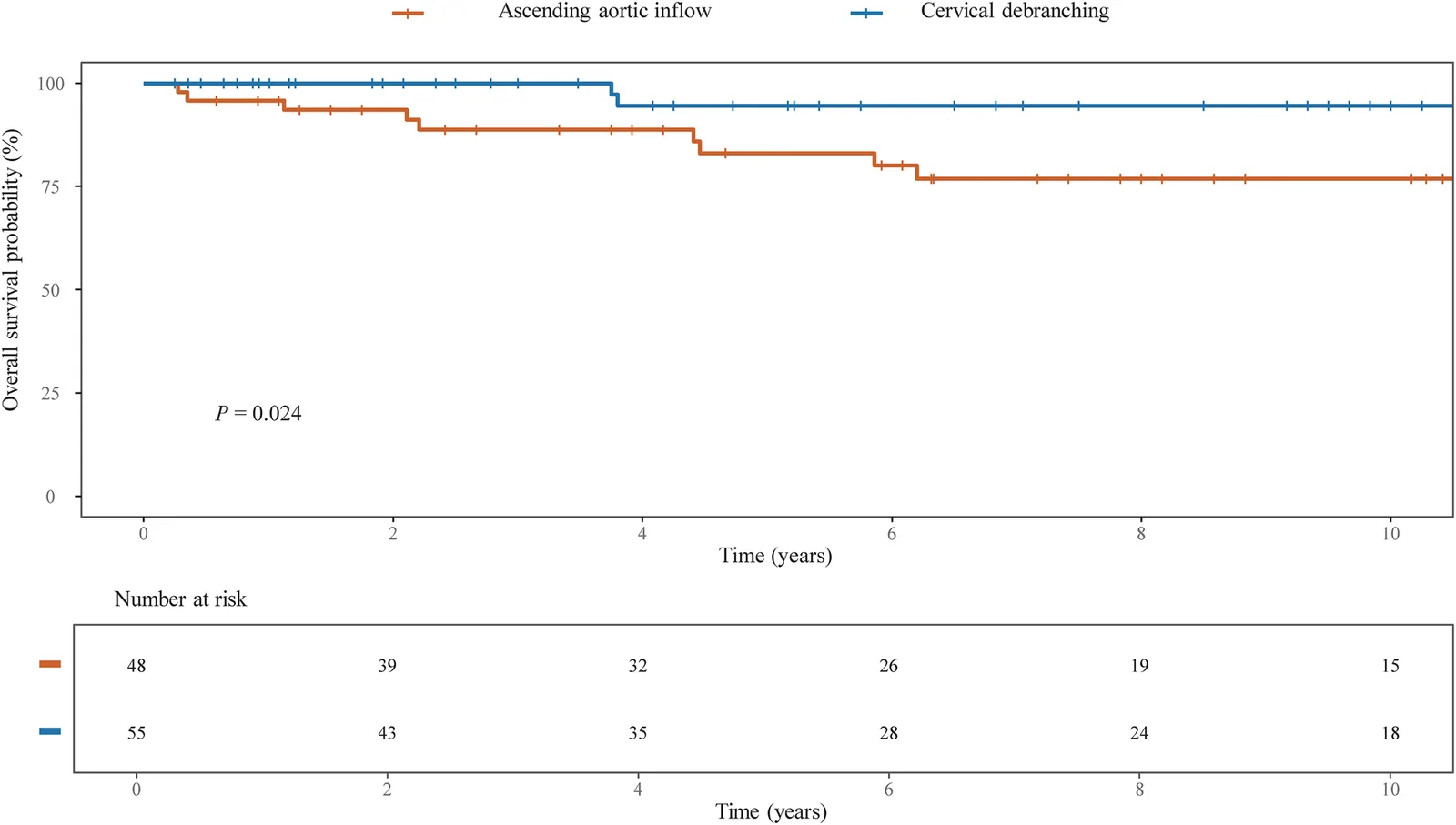
Kaplan–Meier overall survival curves stratified by debranching strategy. Kaplan–Meier overall survival curves comparing patients undergoing ascending aortic inflow reconstruction and cervical debranching. Overall observed survival was higher in the cervical debranching group than in the ascending aortic inflow reconstruction group (log-rank *P* = 0.024). Numbers at risk are shown below the *x*-axis.

The median follow-up duration was 75.8 months (IQR, 29–128 months). As shown in Table 3, 11 patients died during follow-up, corresponding to an overall mortality rate of 10.7%. Deaths occurred at a median of 39 months (IQR, 14–54 months) after HAR. The causes of death were predominantly non-aortic, mainly including stroke and pneumonia. Detailed information on deceased patients is provided in Supplementary Table 2. Furthermore, Kaplan–Meier survival curves indicated that the overall long-term survival of this cohort was well maintained. At the 10-year follow-up mark, 33 patients remained alive and under continuous surveillance (Figure 1). The overall survival rates at 1, 3, 5, and 10 years were 98.0% (95% CI 95.4%–100.0%), 94.6% (95% CI 90.0%–99.3%), 89.0% (95% CI 82.4%–96.1%), and 85.8% (95% CI 78.2%–94.1%), respectively. Stratified analysis demonstrated that the survival rate of the ascending aortic inflow group was significantly lower than that of the cervical debranching group (log-rank *P* = 0.024, Figure 2). However, after excluding patients with type A dissection, the survival difference between the AA inflow and cervical debranching groups was attenuated and no longer statistically significant (log-rank *P* = 0.077).

**Figure 3:**
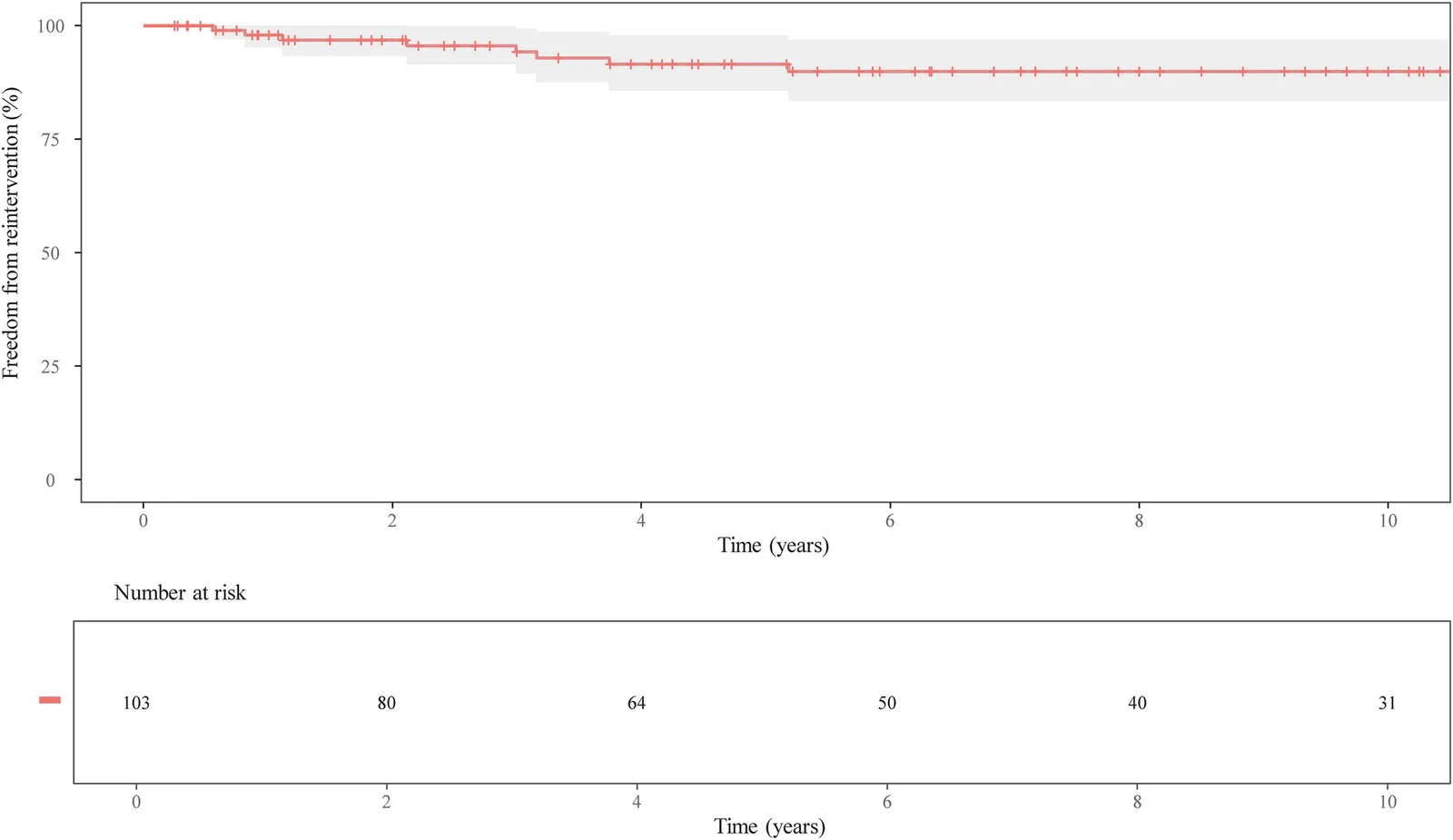
Kaplan–Meier freedom from reintervention curve for the entire cohort after hybrid aortic arch repair. Kaplan–Meier analysis showing freedom from reintervention in the entire cohort during long-term follow-up after hybrid aortic arch repair. The shaded area represents the 95% confidence interval. Numbers at risk are shown below the *x*-axis.

**Figure 4:**
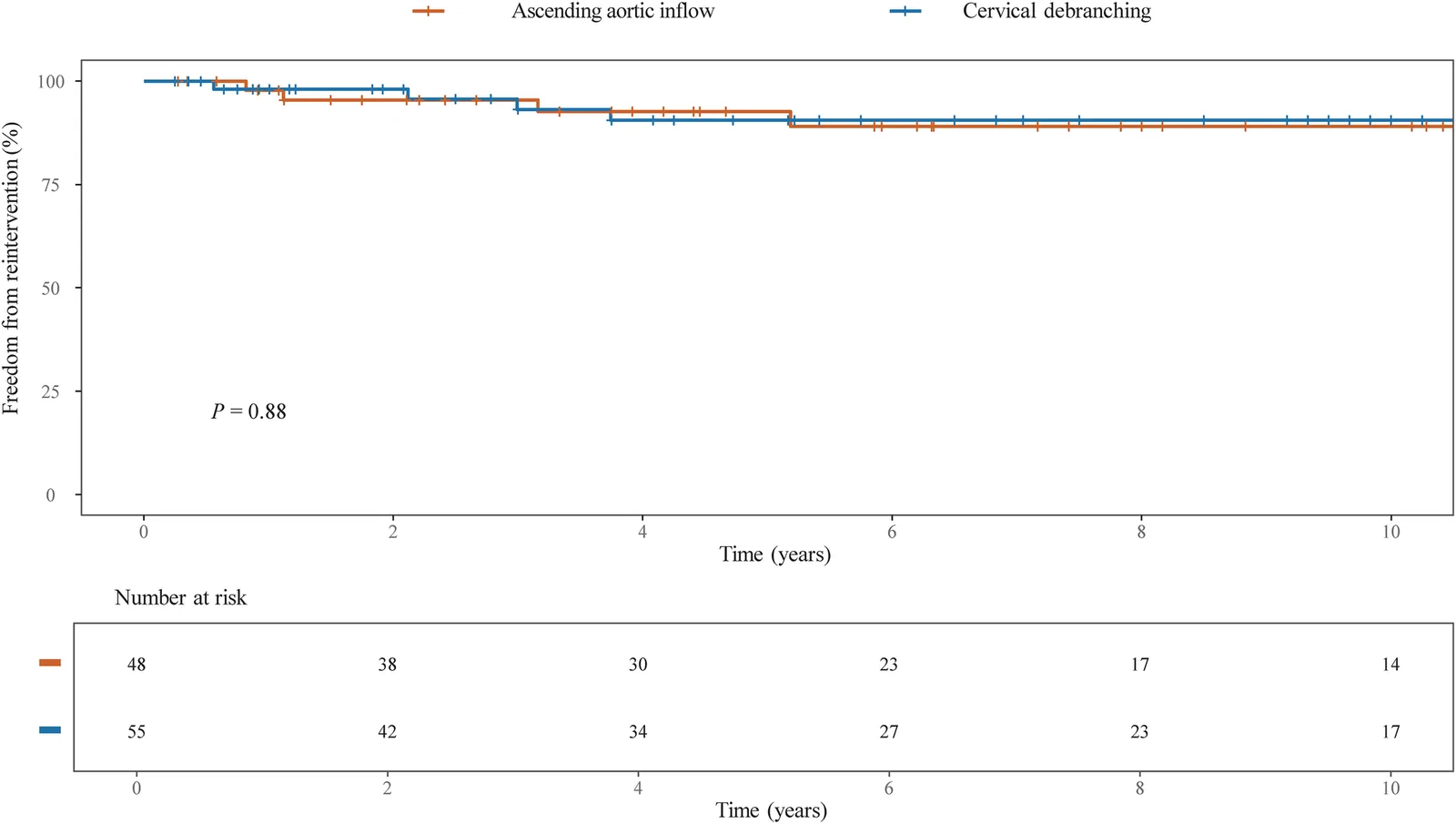
Kaplan–Meier freedom from reintervention curves stratified by debranching strategy. Kaplan–Meier freedom from reintervention curves comparing patients undergoing ascending aortic inflow reconstruction and cervical debranching during follow-up after hybrid aortic arch repair. There was no significant difference in reintervention between the two groups (log-rank *P* = 0.88). Numbers at risk are shown below the *x*-axis.

During the follow-up, 8 patients required secondary major vascular interventions, translating to a reintervention rate of 7.8% and the median time to reintervention was 31 months (IQR, 11.5–41.5 months). Table 3 shows that the primary reasons for reoperation included retrograde type A aortic dissection (RTAD), dilation of the original/new entry tear, graft occlusion, severe branch stent stenosis, and type I endoleak. Specific reintervention details are listed in Supplementary Table 3. Kaplan–Meier analysis confirmed that the overall freedom from reintervention rate remained high throughout the long-term follow-up. The freedom from reintervention rates at 1, 3, 5, and 10 years were 97.9% (95% CI 95.1%–100.0%), 94.2% (95% CI 89.4%–99.3%), 91.5% (95% CI 85.7%–97.8%), and 89.9% (95% CI 83.4%–96.9%), respectively (Figure 3). Moreover, there was no significant difference in this metric between the AA inflow and cervical debranching groups (log-rank *P* = 0.88, Figure 4).

### 3.5 Prognostic factors for long-term mortality

The results of the Cox regression analysis are presented in Table 4. In the univariate analysis, advanced age, AA as the inflow tract, and preoperative renal dysfunction were significantly associated with late mortality. After adjusting for potential confounders in the multivariate model, age (per 1-year increase: HR 1.217, 95% CI 1.086–1.364, *P* < 0.001), AA inflow (HR 11.369, 95% CI 2.069–62.455, *P* = 0.005), and preoperative renal dysfunction (HR 4.318, 95% CI 1.072–17.404, *P* = 0.040) were associated with late mortality in the exploratory multivariable Cox model. Furthermore, given the limited number of late mortality events in this cohort, to prevent overfitting of the multivariate Cox model, this study only incorporated the three core variables with the greatest clinical significance and the most pronounced univariate analysis results into the final model. These findings require further validation in future large-sample studies.

**Table 4:** Univariate and multivariate Cox regression analyses of factors associated with long-term mortality in patients with aortic arch lesions.

Variables	Univariate analysis		Multivariate analysis	
	HR (95% CI)	*P*-value	HR (95% CI)	*P*-value
Age (per 1-year increase)	1.118 (1.031–1.212)	0.007	1.217 (1.086–1.364)	<0.001
Sex (Male vs. Female)	1.427 (0.308–6.617)	0.650	—	—
Inflow source (Ascending aorta vs. Cervical vessels)	4.913 (1.061–22.743)	0.042	11.369 (2.069–62.455)	0.005
Aortic dissection (vs. Non-dissection)	1.786 (0.545–5.853)	0.338	—	—
Hypertension	NE^a^	0.998	—	—
Diabetes mellitus	1.680 (0.491–5.742)	0.408	—	—
Hyperlipidemia	0.717 (0.210–2.451)	0.596	—	—
Coronary artery disease	1.272 (0.372–4.352)	0.701	—	—
Hepatic dysfunction	NE^a^	0.999	—	—
Preoperative renal dysfunction	4.369 (1.158–16.486)	0.030	4.318 (1.072–17.404)	0.040
Prior median sternotomy	1.618 (0.206–12.715)	0.648	—	—

HR, hazard ratio; CI, confidence interval; NE, not estimable.

The multivariate Cox regression model included variables with statistical significance in the univariate analysis (age, inflow source, and preoperative renal dysfunction). Hazard ratio estimates for hypertension and hepatic dysfunction were unstable due to the extremely low number of events or imbalanced distribution; these results should be interpreted with caution. The Schoenfeld residuals test showed that the multivariate model did not violate the proportional hazards assumption (age *P* = 0.55, inflow source *P* = 0.90, renal dysfunction *P* = 0.43, global *P* = 0.74).

a HR not estimable due to complete or quasi-complete separation.

## 4 Discussion

The treatment of aortic arch lesions remains challenging because of the complex anatomy of the arch, the proximity of critical supra-aortic branches, and the frequent coexistence of systemic comorbidities. Conventional open arch replacement often requires cardiopulmonary bypass, deep hypothermic circulatory arrest, and complex cerebral protection, resulting in substantial operative trauma and perioperative risk. These limitations are particularly relevant in elderly patients and those with significant comorbidities. HAR combines open supra-aortic revascularization with endovascular exclusion of the diseased aortic segment, thereby avoiding or reducing the need for circulatory arrest and extensive open arch reconstruction in selected high-risk patients. In this retrospective single-center cohort, we evaluated long-term survival, freedom from reintervention, perioperative complications, and factors associated with late mortality after HAR.

Open surgery has long been the gold standard for aortic arch lesions; however, elderly patients, those requiring reoperations, and those with multiple comorbidities often cannot tolerate traditional open total arch replacement (2, 3, 20, 21). HAR provides a tailored alternative pathway for high-risk populations. Previous studies have reported significant variations in mid- and long-term survival rates following HAR, with some centers documenting 5-year overall survival rates of approximately 59%–85% and 10-year rates of 47%–74% (5, 15, 17, 22–24). In this study, the 10-year overall survival rate was 85.8%, and the reintervention rate was 7.8%, which is highly consistent with mainstream literature. Recently, Vekstein et al. (17) demonstrated that the 10-year aorta-specific survival rate of HAR cohorts could reach 84%.

The average age of the patients in our cohort was 72 years, and the vast majority harbored multiple comorbidities. They represent a high-risk population for traditional open surgery, consistent with the indication principles of hybrid surgery (3). Regarding pathology types, intramural hematoma accounted for the highest proportion, followed by aortic dissection. This distribution differs from prior studies (17, 18) dominated primarily by dissections and aneurysms, suggesting that hybrid surgery is effective not only for severe lesions like dissections and aneurysms but also serves as a feasible treatment option for non-dissection lesions such as intramural hematomas and penetrating ulcers. Particularly for patients with non-dissection lesions who respond poorly to medical therapy and face a risk of progression, hybrid surgery can effectively halt disease advancement and improve prognosis.

During the 15-year study period, the management of aortic arch disease evolved considerably. Total endovascular arch repair, including branched or fenestrated arch endografts, *in situ* fenestration, chimney or parallel graft techniques, and physician-modified stent-grafts, has emerged as an alternative for selected high-risk patients (25). These approaches may reduce the need for sternotomy or supra-aortic debranching and may be particularly useful in patients with suitable landing zones and favorable arch anatomy. However, their widespread application remains limited by anatomical constraints, device availability, technical complexity, regulatory approval, risk of stroke or endoleak, and limited long-term durability data (25, 26). During much of our study period, dedicated branched arch devices were not routinely available in our center. Therefore, hybrid aortic arch repair remained a practical and adaptable strategy for patients with complex arch pathology who were unsuitable for conventional open repair. Future comparative studies are needed to define the relative roles of open, hybrid, and total endovascular arch repair in anatomically comparable patient populations.

An important finding of this study was the association between debranching strategy and observed long-term survival. Kaplan–Meier analysis showed higher observed survival in the cervical debranching group than in the ascending aortic inflow group. In the exploratory multivariable Cox model, ascending aortic inflow was also associated with late mortality. However, this finding should not be interpreted as evidence that Zone 0 or ascending aortic inflow reconstruction is intrinsically harmful. Rather, it likely reflects the greater complexity of proximal aortic disease in patients requiring this strategy. As shown in Table 1, all patients with type A aortic dissection were treated in the ascending aortic inflow group, and many required more invasive concomitant procedures. After excluding patients with type A dissection, the survival difference between the two groups was attenuated and no longer statistically significant, supporting the possibility that the observed survival gap was largely driven by baseline disease complexity. Therefore, the comparison between debranching strategies should be considered hypothesis-generating and interpreted with caution. Conversely, the cervical debranching group predominantly consisted of patients with isolated aneurysms, IMH, or chronic type B dissections, completely circumventing thoracic entry. Previous studies (8, 24, 27–29) have corroborated this phenomenon that patients requiring Zone 0 interventions inherently possess a more severe disease phenotype and inferior long-term physiological reserves. Therefore, this outcome cannot be simplistically interpreted as a pure causal effect of the surgical procedure itself. These conclusions require further validation through large-sample, multicenter studies with more robust adjustments for confounding factors. In addition, physician-modified stent-grafts and 3D printing-assisted endovascular aortic repair have evolved as precise, patient-specific strategies to manage complex arch and thoracoabdominal lesions. These technologies allow for the creation of fenestrated or branched grafts tailored to unique anatomical constraints, thereby optimizing the proximal landing zone without the need for high-risk surgical inflow maneuvers. As highlighted by Kayan et al. (30) and Zhu et al. (31), 3D printing-assisted techniques significantly enhance procedural planning and accuracy in endovascular repair, providing a viable and less invasive therapeutic perspective for high-risk cohorts where traditional Zone 0 inflow may carry prohibitive mortality risks.

Advanced age is a classic risk factor for poor prognosis in patients with complex aortic lesions. This study found that as the age of surgical patients increased, so did their mortality risk. This finding aligns with previous studies, highlighting that elderly patients possess diminished organ functional reserves, poorer perioperative tolerance, and limited long-term recovery capabilities (3, 21).

Preoperative renal dysfunction was also independently associated with the risk of late mortality in this study. Renal impairment not only reflects a compromised overall baseline status but also serves as a clinical manifestation of extensive systemic atherosclerosis and depleted physiological reserve. It significantly amplifies the risks of contrast-induced nephropathy post-TEVAR and long-term cardiovascular death (4, 32, 33). Thus, for these patients, intense focus must be placed on contrast exposure control, hemodynamic optimization, and renal function monitoring during the perioperative period.

The neurological outcomes of this study are encouraging. Although frequent intra-arch wire manipulation and clamping of the supra-aortic vessels resulted in a 7.8% incidence of procedure-related stroke, which was consistent with the 6%–11% range reported in most HAR registries (5, 24), the incidence of spinal cord ischemia in our cohort was zero. This absence of spinal cord ischemia is highly likely attributable to our aggressive approach to LSA revascularization. Routine preservation of the LSA prevents the occlusion of the critical anterior spinal artery collateral network, which aligns with the theoretical basis of current vascular surgical guidelines that strongly recommend LSA reconstruction during TEVAR (3, 23).

In addition, no patients in this study experienced severe bilateral upper limb blood pressure asymmetry postoperatively, indicating that the extra-anatomic bypass methods utilized provided effective supra-aortic revascularization without inducing upper limb ischemia. This success is closely related to the selection of artificial grafts and anastomotic techniques during surgery. Ensuring that the vessels are free of tension and kinking during anastomosis effectively enhances the patency of the bypass grafts and reduces the occurrence of upper limb blood pressure asymmetry.

The overall reintervention rate in this cohort was 7.8%, which is relatively low compared to several previous reports (14, 17, 23). Kaplan–Meier freedom from reintervention analysis revealed no significant difference between the two bypass strategies, suggesting that despite variances in overall survival, the risk of secondary interventions does not synchronously increase. This indicates that once the early high-risk postoperative period is surpassed, the anatomical durability of both bypass architectures is equally reliable. Additionally, we recorded 2 cases of retrograde type A aortic dissection (RTAD) post-HAR, compelling subsequent open total arch replacement combined with frozen elephant trunk implantation. RTAD is the most lethal complication of HAR, primarily triggered by the radial expansile force exerted by the stent deployed within a diseased, non-replaced ascending aorta. This outcome underscores that long-term management following HAR must not only focus on the arch repair itself but also involve continuous surveillance of the evolution of residual aortic lesions.

Renal impairment and stroke were the most common postoperative morbidities we observed. Postoperative renal dysfunction after HAR is likely multifactorial. Large-diameter thoracic stent-graft implantation may trigger systemic inflammatory activation and post-implantation syndrome, which may contribute to transient organ dysfunction (34). Iodinated contrast exposure during preoperative imaging, intraoperative angiography, and postoperative surveillance may increase the risk of contrast-associated acute kidney injury, particularly in elderly patients and those with pre-existing renal impairment (35). In addition, extensive manipulation of the aortic arch and descending thoracic aorta may promote atheroembolism or microembolization, while perioperative hypotension, blood loss, transfusion, prolonged operative time, and renal hypoperfusion may further aggravate renal injury (25). These mechanisms may be particularly relevant in patients undergoing more invasive proximal aortic procedures. Therefore, renal protection after HAR should include not only attention to inflammatory responses but also minimization of contrast volume, maintenance of stable hemodynamics, avoidance of nephrotoxic agents, and close monitoring of renal function. Because stroke represented both a major complication and a leading cause of mortality, optimal perioperative neuroprotection remains essential during aortic arch surgery. Pneumonia also contributed heavily to postoperative deaths. This highlights that maintaining respiratory function, encouraging early mobilization, and preventing infections are equally vital for the long-term survival of elderly patients. The evolution of TEVAR devices during the study period should also be considered when interpreting the long-term outcomes. Improvements in delivery systems, conformability, and device availability may have influenced procedural planning and outcomes over time. However, because device selection was individualized and the number of patients treated with each device was limited, this study was not designed to compare outcomes among different endograft brands.

Overall, long-term outcomes after HAR are driven not solely by the success of the aortic repair, but by a complex interplay of advanced age, underlying organ reserve, and the management of chronic comorbidities.

## 5 Study limitations

Several limitations should be acknowledged. First, this was a retrospective, single-center study with a relatively small sample size, and selection bias and survival bias cannot be excluded. Second, the comparison between ascending aortic inflow and cervical debranching was affected by confounding by indication, because the choice of strategy was strongly influenced by proximal aortic disease complexity, landing-zone requirements, and the need for concomitant procedures. Although a sensitivity analysis excluding patients with type A dissection was performed, residual confounding remains possible. Propensity-score matching was considered but not performed because of the limited sample size, the low number of late deaths, and the complete separation of type A dissection between groups, which would have resulted in substantial loss of sample size and unstable estimates. Third, only 11 late deaths occurred, resulting in a low event-per-variable ratio in the multivariable Cox model. Therefore, the Cox regression findings should be considered exploratory, and the estimates, particularly for ascending aortic inflow, may be unstable, as reflected by the wide confidence intervals. Fourth, device selection evolved during the 15-year study period, and the study was not designed to compare outcomes among different TEVAR devices. Finally, the database did not capture several long-term patient-centered outcomes, such as aorta-related mortality, functional recovery, or quality of life. Larger multicenter prospective registries with standardized imaging follow-up and longer observation are needed to validate these findings.

## 6 Conclusion

HAR is a practical treatment option for selected high-risk patients with aortic arch disease and was associated with favorable mid- to long-term outcomes in this single-center cohort. No spinal cord ischemia occurred, and the overall freedom from reintervention remained high during follow-up. Older age, ascending aortic inflow, and preoperative renal dysfunction were associated with late mortality in an exploratory multivariable Cox model. The higher observed mortality in the ascending aortic inflow group likely reflects greater proximal aortic disease complexity rather than a direct causal effect of the bypass strategy itself. Cervical debranching may be a reasonable option in anatomically suitable patients with an adequate proximal landing zone. For patients requiring ascending aortic inflow reconstruction, careful perioperative management, renal protection, and lifelong imaging surveillance remain essential.

## Data Availability

The original contributions presented in the study are included in the article/Supplementary Material, further inquiries can be directed to the corresponding authors.
