# Supplementary material for: Long-term outcomes and prognostic factors of hybrid aortic arch repair: a 15-year cohort study
Source: Front Cardiovasc Med. 2026 Sep 4;13:1933618. doi: 10.3389/fcvm.2026.1933618 (PMC13585604; doi:10.3389/fcvm.2026.1933618)
Supplement: Supplementary file 1 [file Table1.docx]

**Supplementary Table 1. Thoracic endografts used during the study period**

| Device Model / Name | Manufacturer | Period of use | Relative use in cohort | Notes |
| --- | --- | --- | --- | --- |
| Valiant^™^ (Captivia^™^ / Navion^™^) | Medtronic | Early and selected later cases | Less frequent | Used in selected cases; limited by device availability and selection options |
| GORE^®^ TAG^®^ / Conformable TAG^®^ | W. L. Gore & Associates | Throughout study period, more frequent later | Common | Became one of the predominant devices in later practice |
| E-vita Thoracic / E-xl | JOTEC / Artivion | Early phase | Infrequent | Limited domestic use and device-specific anatomical applicability |
| Zenith^®^ Alpha^™^ / Zenith^®^ TX2^®^ | Cook Medical | Early phase | Infrequent | Limited domestic use and device-specific anatomical applicability |
| Ankura^™^ | Lifetech Scientific | Early and later phase | Common later | Frequently used in later practice |
| Hercules^™^ / Castor^™^ | MicroPort Scientific | Early and later phase | Common later | Frequently used in later practice |
