# Supplementary material for: Long-term outcomes and prognostic factors of hybrid aortic arch repair: a 15-year cohort study
Source: Front Cardiovasc Med. 2026 Sep 4;13:1933618. doi: 10.3389/fcvm.2026.1933618 (PMC13585604; doi:10.3389/fcvm.2026.1933618)
Supplement: Supplementary file 2 [file Table2.docx]

Supplementary Table 2. Clinical profiles of 11 patients with aortic arch lesions who died during follow-up after hybrid procedures

| Patient No. | Sex | Age (years) | Preoperative diagnosis | Hybrid procedure performed | Time to death after HAR (months) | Cause of death |
| --- | --- | --- | --- | --- | --- | --- |
| 1 | M | 73 | Type A aortic dissection | Ascending aorta replacement + AA-IA-LCCA-LSA bypass + TEVAR | 75 | Pneumonia |
| 2 | M | 77 | Type A aortic dissection | Bentall procedure + AA-IA, AA-LCCA, and AA-LSA bypasses + TEVAR | 70 | Cerebral hemorrhage |
| 3 | F | 81 | Aortic intramural hematoma | Ascending aorta replacement + AA-IA-LCCA-LSA bypass + TEVAR | 25 | Pneumonia |
| 4 | M | 79 | Aortic arch aneurysm | RSA-LCCA-LSA bypass + TEVAR | 39 | Cerebral infarction |
| 5 | M | 76 | Aortic intramural hematoma | Ascending aorta replacement + AA-IA-LCCA-LSA bypass + TEVAR | 54 | Cerebral infarction |
| 6 | M | 73 | Penetrating aortic ulcer of the aortic arch | RSA-LSA bypass + TEVAR | 3 | Typhoid infection |
| 7 | F | 77 | Aortic intramural hematoma | Ascending aorta replacement + AA-IA-LCCA-LSA bypass + TEVAR | 26 | Cerebral infarction |
| 8 | M | 79 | Aortic arch aneurysm | RSA-LCCA-LSA bypass + TEVAR | 45 | Myocardial infarction |
| 9 | M | 76 | Type B aortic dissection | RSA-LSA bypass + TEVAR | 44 | Cerebral infarction |
| 10 | M | 83 | Type A aortic dissection | Bentall procedure + AA-IA, AA-LCCA, and AA-LSA bypasses + TEVAR | 4 | Cerebral infarction |
| 11 | M | 81 | Type A aortic dissection | Bentall procedure + AA-IA, AA-LCCA, and AA-LSA bypasses + TEVAR | 14 | Pneumonia |

TEVAR, thoracic endovascular aortic repair; RSA, right subclavian artery; LSA, left subclavian artery; LCCA, left common carotid artery; AA, ascending aorta; IA, innominate artery; M, male; F, female.
