# Supplementary material for: Long-term outcomes and prognostic factors of hybrid aortic arch repair: a 15-year cohort study
Source: Front Cardiovasc Med. 2026 Sep 4;13:1933618. doi: 10.3389/fcvm.2026.1933618 (PMC13585604; doi:10.3389/fcvm.2026.1933618)
Supplement: Supplementary file 3 [file Table3.docx]

Supplementary Table 3. Details of 8 patients requiring reintervention during follow-up after hybrid procedures

| Patient No. | Sex | Age (years) | Preoperative diagnosis | Initial procedure | Indication for reintervention | Time to reintervention (months) | Reintervention procedure | Outcome |
| --- | --- | --- | --- | --- | --- | --- | --- | --- |
| 1 | M | 76 | Type B aortic dissection | RSA-LSA bypass + TEVAR | Retrograde type A dissection | 45 | Ascending aorta and total arch replacement + FET | Died (cerebral infarction) |
| 2 | M | 63 | Type B aortic dissection | RSA-LCCA-LSA bypass + TEVAR | Retrograde type A dissection | 7 | Ascending aorta and total arch replacement + FET | Discharged |
| 3 | F | 71 | Type A aortic dissection | Ascending aorta replacement + AA-IA-LCCA-LSA bypass + TEVAR | Dilated entry tear in descending aorta | 62 | TEVAR | Discharged |
| 4 | M | 69 | Type A aortic dissection | Ascending aorta replacement + AA-IA-LCCA-LSA bypass + TEVAR | Dilated entry tear in descending aorta | 13 | TEVAR | Discharged |
| 5 | M | 73 | Intramural hematoma | Ascending aorta replacement + AA-IA-LCCA-LSA bypass + TEVAR | New entry tear in descending aorta | 10 | TEVAR | Discharged |
| 6 | M | 72 | Type B aortic dissection | RSA-LCCA-LSA bypass + TEVAR | Prosthetic graft occlusion | 38 | Replacement of previous LCCA-LSA bypass graft | Discharged |
| 7 | F | 81 | Type B aortic dissection, aortic diverticulum | RSA-LSA bypass + TEVAR (branch stent-graft) | Severe branch stent stenosis | 26 | Balloon angioplasty of the stent-graft | Discharged |
| 8 | M | 75 | Aortic arch aneurysm | RSA-LSA bypass + TEVAR | Type I endoleak | 36 | AA-IA-LCCA bypass + TEVAR | Discharged |

TEVAR, thoracic endovascular aortic repair; FET, frozen elephant trunk; RSA, right subclavian artery; LSA, left subclavian artery; LCCA, left common carotid artery; AA, ascending aorta; IA, innominate artery; M, male; F, female.
